# Mixed Autoimmune Hemolytic Anemia: A Systematic Review of Epidemiology, Clinical Characteristics, Therapies, and Outcomes

**DOI:** 10.1002/ajh.27721

**Published:** 2025-05-20

**Authors:** Jeremy W. Jacobs, Sheharyar Raza, Landon M. Clark, Laura D. Stephens, Elizabeth S. Allen, Jennifer S. Woo, Rachel Lane Walden, Cristina A. Figueroa Villalba, Christopher A. Tormey, Caroline G. Stanek, Brian D. Adkins, Evan M. Bloch, Garrett S. Booth

**Affiliations:** ^1^ Department of Pathology, Microbiology & Immunology Vanderbilt University Nashville Tennessee USA; ^2^ Laboratory Medicine and Pathobiology University of Toronto Toronto Ontario Canada; ^3^ Canadian Blood Services Medical Affairs and Innovation Toronto Ontario Canada; ^4^ Vanderbilt University School of Medicine Nashville Tennessee USA; ^5^ Department of Pathology University of California San Diego La Jolla California USA; ^6^ Department of Pathology City of Hope Comprehensive Cancer Center Irvine California USA; ^7^ Annette and Irwin Eskind Family Biomedical Library and Learning Center, Vanderbilt University Nashville Tennessee USA; ^8^ Department of Laboratory Medicine Yale School of Medicine New Haven Connecticut USA; ^9^ Department of Pathology University of Alabama at Birmingham Birmingham Alabama USA; ^10^ Department of Pathology University of Texas Southwestern Medical Center Dallas Texas USA; ^11^ Division of Transfusion Medicine, Department of Pathology Johns Hopkins University Baltimore Maryland USA

**Keywords:** hemolysis, hemolytic anemia, mixed AIHA, mixed autoimmune hemolysis, mixed autoimmune hemolytic anemia, mixed‐type autoimmune hemolytic anemia

## Abstract

Mixed autoimmune hemolytic anemia (AIHA) is a rare and clinically complex hematologic disorder defined by the simultaneous presence of both warm and cold autoantibodies, resulting in severe and often treatment‐resistant hemolysis. Due to variability in diagnostic criteria and limited data, a comprehensive understanding of its epidemiology, clinical characteristics, and management remains incomplete. To address these gaps, we performed a systematic literature review employing stringent diagnostic criteria to evaluate epidemiologic patterns, clinical features, and therapeutic outcomes. Our analysis included 81 patients identified across 35 studies, revealing a median age of 45 years and a notable female predominance (2.25:1). Autoimmune diseases constituted the most frequent underlying etiology, followed by hematologic malignancies and infections. Patients exhibited significant anemia, with median nadir hemoglobin levels reaching 5.6 g/dL. Corticosteroids represented the most common therapeutic intervention; however, only 43% of patients achieved remission, while 37% experienced chronic hemolysis, and mortality reached 11%. Many patients required multiple lines of therapy, including rituximab and cytotoxic agents, highlighting the disease's refractory nature and management complexity. The substantial variability in diagnostic and therapeutic approaches emphasizes an urgent need for standardized diagnostic criteria, earlier integration of combination therapies, and exploration of innovative treatment modalities. Future prospective, multicenter studies are essential to refine disease recognition, optimize therapeutic strategies, and ultimately improve patient outcomes in mixed AIHA.

## Graphical abstract

Mixed autoimmune hemolytic anemia (AIHA) is a rare condition characterized by the presence of both warm and cold autoantibodies, often leading to severe, treatment‐resistant hemolysis. In this systematic review of 81 patients across 35 studies, the median age was 45 years with a notable female predominance. Autoimmune diseases constituted the most frequent underlying etiology, followed by hematologic malignancies and infections. Median nadir hemoglobin was 5.6 g/dL. Corticosteroids were the most common therapeutic intervention, although more than half of patients required multiple lines of treatment. Only 43% of patients achieved remission, while 37% experienced chronic hemolysis and 11% died. These findings highlight the urgent need for standardized diagnostics, early use of combination therapies, and investigation of novel treatments.

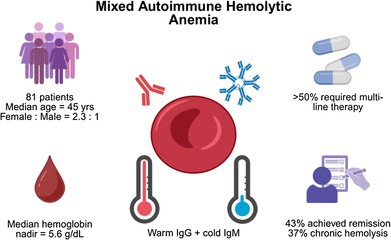

## 1 Introduction

Autoimmune hemolytic anemia (AIHA) is a rare, acquired disorder characterized by the premature destruction of autologous red blood cells (RBCs) due to autoantibody‐mediated hemolysis [1, 2, 3, 4, 5]. The etiology of AIHA is multifaceted [6]. Cases are typically considered either primary or secondary to conditions such as autoimmune diseases, infections, malignancies, or drug exposure [7, 8]. AIHA may be categorized based on the optimal thermal reactivity of the autoantibodies involved, resulting in distinct subtypes including warm AIHA, cold AIHA (i.e., cold agglutinin disease or cold agglutinin syndrome), mixed AIHA, and paroxysmal cold hemoglobinuria (PCH) [7, 9, 10, 11, 12, 13]. Among these, warm AIHA is the most prevalent, accounting for over two‐thirds of adult cases, whereas mixed AIHA, although less common, remains clinically significant [14].

Mixed AIHA is characterized by the presence of both warm (IgG)‐ and cold (IgM)‐reactive autoantibodies, resulting in a complex and often treatment‐resistant clinical presentation [15, 16, 17, 18]. Reports suggest that mixed AIHA accounts for fewer than 10% of AIHA cases [3, 19, 20, 21], though its true incidence is difficult to ascertain due to inconsistencies in diagnostic criteria across studies [20, 22]. Some studies have proposed that mixed AIHA imparts a more severe clinical course, often necessitating multiple lines of therapy and leading to increased transfusion dependence [17, 18]. However, the rarity of the condition—coupled with the variability in laboratory definitions and diagnostic methodologies—continues to hinder a comprehensive understanding of its epidemiology and optimal management [12].

Previous literature reviews on mixed AIHA have been limited by inconsistent case definitions and methodological challenges [20, 23]. Many studies have diagnosed mixed AIHA using broad or non‐specific diagnostic criteria, such as a positive direct antiglobulin test (DAT) for both IgG and complement (C3), without further confirmatory testing. Critical assessments, including elution studies to verify warm autoantibodies and thermal amplitude testing to identify pathologic cold autoantibodies, are often absent. To address these limitations, we conducted a systematic review of the available literature on mixed AIHA using strict diagnostic criteria published by the British Society of Hematology and the First International Consensus Meeting [19, 24], aiming to refine our understanding of its epidemiology, clinical presentation, and responses to treatment.

## 2 Methods

### 2.1 Objective

The aim of this systematic review was to assess the epidemiology, clinical features, laboratory findings, therapeutic strategies, and outcomes for patients with mixed AIHA using strict diagnostic criteria.

### 2.2 Registration

This study was performed in accordance with the Preferred Reporting Items for Systematic review and Meta‐Analysis Protocols (PRISMA‐P) [25]. In accordance with the guidelines, this systematic review protocol was submitted for registration with the International Prospective Register of Systematic Reviews (PROSPERO) on October 01, 2024 and was registered on October 12, 2024 (CRD42024596204). As no protected or identifiable information was accessed or reported, this study was exempt from review by the institutional review board.

### 2.3 Search Strategy

A medical subject headings (MeSH) analysis of known key articles provided by the research team was performed, and pilot searches were performed in each database. An iterative process was used to translate and refine the pilot searches. To maximize sensitivity, the formal search used controlled vocabulary terms and synonymous free‐text words. Searches were conducted in PubMed (NLM), Embase (Elsevier), Web of Science (Clarivate), Scopus (Elsevier), and Cochrane Central Register of Controlled Trials (Wiley) on September 30, 2024. No date or language limits were applied. A lateral search of citations in eligible studies and relevant review articles was also performed. Searches were re‐run prior to the final analysis to identify any studies published after the initial search. The PubMed search strategy is listed below, and the comprehensive search strategy is outlined in the Supporting Information: (pages 6–8).

(“Anemia, Hemolytic, Autoimmune”[Mesh] OR AIHA[tiab] OR autoimmune haemolysis[tiab] OR autoimmune hemolysis[tiab] OR autoimmune haemolytic anaemia*[tiab] OR autoimmune haemolytic anemia*[tiab] OR autoimmune hemolytic anaemia[tiab] OR autoimmune hemolytic anemia*[tiab] OR cold agglutinin disease*[tiab] OR cold antibody disease*[tiab] OR cold antibody haemolytic anaemia*[tiab] OR cold antibody haemolytic anemia*[tiab] OR cold antibody hemolytic anaemia*[tiab] OR cold antibody hemolytic anemia*[tiab] OR immune mediated anaemia*[tiab] OR immune mediated anemia*[tiab]) AND ((Mixed[tiab] OR combined[tiab]) OR (warm[tiab] AND cold[tiab]) OR (IgM[tiab] AND IgG[tiab]) OR (immunoglobulin M[tiab] AND immunoglobulin G[tiab])).

### 2.4 Data Management

Search results were managed in EndNote 21 [www.endnote.com]. Title and abstract screening, full text review, and data extraction were performed in Covidence [www.covidence.org] and Microsoft Excel. Review author pairs (J.W.J., S.R., C.G.S., J.S.W., G.S.B., L.M.C.) independently screened the titles and abstracts yielded by the search against the inclusion criteria. Full reports were obtained for all titles that appeared to meet the inclusion criteria or where there was any uncertainty. Review author pairs then independently screened the full text reports for inclusion or exclusion. Discrepancies were adjudicated by a review author not involved in the discrepancy.

### 2.5 Inclusion and Exclusion Criteria

Eligible studies included randomized and non‐randomized trials, cross‐sectional studies, retrospective and prospective cohort analyses, case–control analyses, case series, and case reports. Reviews were assessed for additional cases not identified in our primary literature search. There was no time limit, and articles in any language were included if they could be translated using a Web‐based translation system (Google Translate). Studies were required to present data on patients with mixed AIHA, categorized as either “definite” or “possible/probable” cases. We used guidance from both the British Society of Haematology and the First International Consensus Meeting to inform how we defined mixed AIHA (Table 1) [19, 24]. Cases lacking sufficient laboratory data were excluded.

**TABLE 1:** Diagnostic criteria for mixed AIHA.

Hemolytic anemia	*Anemia* Hemoglobin and/or hematocrit below the local established reference range *Hemolysis* ≥ 2 of: ○ Elevated indirect bilirubin ○ Elevated lactate dehydrogenase ○ Decreased haptoglobin ○ Elevated urobilinogen ○ Hemoglobinuria ○ Increased spherocytes on peripheral blood smear
Mixed autoimmune hemolytic anemia	*All cases* Positive DAT for IgG and complement *Definite* Positive elution study ○ Either a pan‐reactive eluate or an eluate with an antibody with apparent specificity for an antigen expressed by the patient (e.g., auto‐anti‐e) OR RBC transfusion has not occurred in the prior 3 months, the patient has no known alloantibodies, and the IAT is pan‐reactive or demonstrates an autoreactive pattern with AHG AND A pathologic cold agglutin is present ○ Cold agglutinin that reacts between 0°C and < 20°C as well as ≥ 30°C *Possible or probable* If elution and IAT results are not reported: ○ RBC transfusion has not occurred in the prior 3 months and all other criteria are met OR ○ RBC transfusion has occurred in the previous 3 months, but the IAT is pan‐reactive If the temperature requirements for cold agglutinin testing are not reported ○ Evidence of RBC agglutination with a cold agglutinin titer ≥ 64

Abbreviations: AHG, antihuman globulin; DAT, direct antiglobulin test; IAT, indirect antiglobulin test; RBC, red blood cell.

### 2.6 Clinical Diagnostic Criteria

A diagnosis (possible/probable or definite) of mixed AIHA requires demonstration of AIHA [anemia (hemoglobin and/or hematocrit below the established reference range) secondary to an immune‐mediated cause involving both warm and cold autoantibodies].

All cases (probable/possible and definite) required the hemolytic component to comprise at least two of: elevated indirect bilirubin; elevated lactate dehydrogenase; decreased haptoglobin; elevated urobilinogen; hemoglobinuria; and increased spherocytes on peripheral blood smear.

The immune component must have comprised a positive direct antiglobulin test (DAT) with both IgG and complement bound to the red cell surface via any standard method (standard tube methods, column agglutination, solid phase red cell adherence). To be classified as a “definite” case of mixed‐type AIHA required demonstration of IgG bound to the RBC surface in the DAT and a positive elution study (i.e., either a pan‐reactive eluate or an eluate with an antibody with apparent specificity for an antigen expressed by the patient, e.g., auto‐anti‐e) with a concomitant pathologic cold agglutin. If the elution was not reported, the case could still be considered “definite” if RBC transfusion had not occurred in the prior 3 months, the patient had no known alloantibodies, and the indirect antiglobulin test (IAT) was pan‐reactive or demonstrated an autoreactive pattern with anti‐human globulin (AHG). If elution and IAT results were not reported, the case could be considered “possible or probable” if RBC transfusion had not occurred in the prior 3 months. If RBC transfusion had occurred in the previous 3 months but the IAT was pan‐reactive, the case was considered “possible or probable.”

Detection of a pathologic cold agglutinin required a DAT positive for C3 with a cold agglutinin that reacted between 0°C and ≤ 20°C as well as ≥ 30°C. If a cold agglutinin was suggested to be present (e.g., via a DAT positive for C3 with concomitant RBC agglutination) but the temperature requirements were not reported, the case could still be included as “possible or probable” if the titer was ≥ 64.

If no testing was available to illustrate the presence of both warm and cold autoantibodies, or if sufficient testing was performed demonstrating that the cold agglutinin did not react ≥ 30°C, the case was excluded.

For studies using the same data set, only discrete and unrelated values were extracted from each study. If we were unable to determine overlapping data, only the larger of the two studies was included.

### 2.7 Data Collection and Synthesis

Two authors independently extracted data on demographics, AIHA risk factors, treatments, RBC transfusion requirements, laboratory parameters, and patient outcomes. Descriptive analyses were conducted using Prism (GraphPad Software, La Jolla, CA, USA). Means and standard deviations were reported for normally distributed data, while medians and interquartile ranges (IQR) were used for non‐normally distributed variables. Statistical significance was determined using *t*‐tests for means or Mann–Whitney tests for medians; Fisher's exact test was used to compare proportions. A *p* value < 0.05 was considered statistically significant.

## 3 Results

### 3.1 Literature Search Results

**FIGURE 1:**
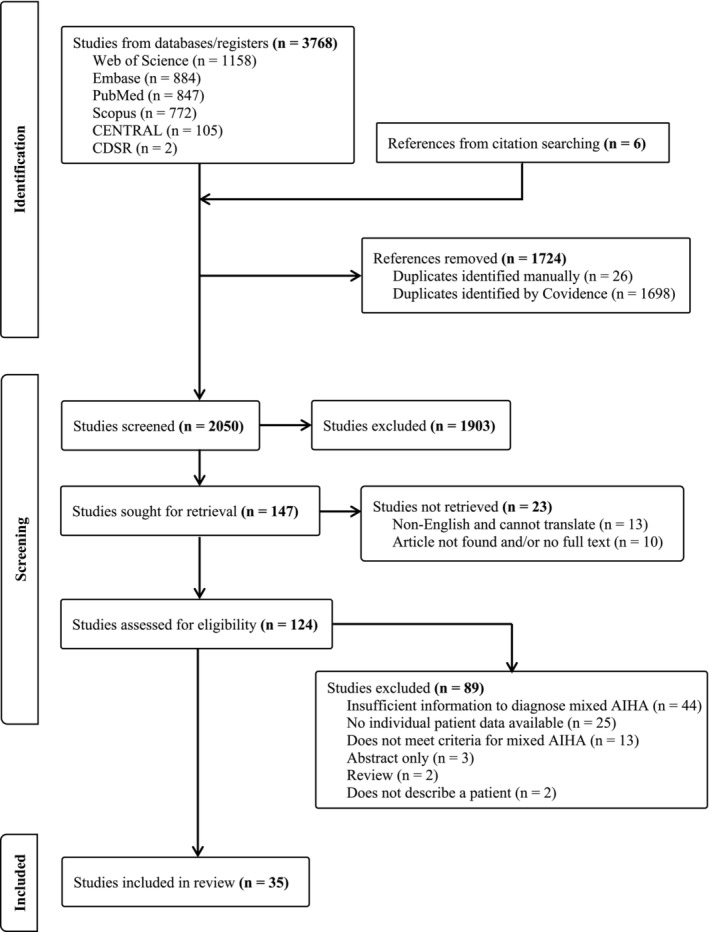
PRISMA flow diagram for systematic review.

The primary literature search identified 3774 studies, of which 35 met the inclusion criteria, encompassing a total of 81 patients (Figure 1, Table S1). Among these, 81.5% (66/81) were classified as “definite” mixed AIHA and 18.5% (15/81) were categorized as “possible or probable.” Notably, 44.4% (36/81) of cases were reported in just two studies [16, 26]. Cases included in the analysis were published between 1975 and 2024 (Figure S1).

### 3.2 Patient Demographics and Associated Conditions

The median age of patients was 45 years (IQR: 24–67) (Table 2), with a female‐to‐male ratio of 2.25:1. There was no difference (*p* = 0.35) in the median age of females [45 years (IQR: 24–67 years), 95% CI 34–63 years] and males [41 years (IQR: 17–61 years), 95% CI: 25–59 years]. When limited to individuals with definite mixed AIHA, the median age was 45 years (IQR: 25–64 years) with a 2.3:1 female‐to‐male ratio. Autoimmune diseases were the most commonly associated conditions (35.8%, 29/81), followed by malignancies (21.0%, 17/81) and infections (12.3%, 10/81) (Table 3); 22.2% (18/81) of patients had no reported condition potentially associated with the development of mixed AIHA.

**TABLE 2:** Demographics of included patients (*n* = 81).

Age	Median (IQR)
All	45 years (24–67 years)
Definite	45 years (25–64 years)
Possible or probable	53 years (20–72 years)
Age groups	No. patients (% of total)
0–9 years	7 (9)
10–19 years	7 (9)
20–29 years	13 (16)
30–39 years	9 (11)
40–49 years	8 (10)
50–59 years	8 (10)
60–69 years	13 (16)
70–79 years	12 (15)
80–89 years	3 (4)
Not reported	1 (1)
Sex	No. patients (% of total)
All	81 (100)
Female	54 (67)
Male	24 (30)
Not reported	3 (4)
Definite	66 (81)
Female	44 (67)
Male	19 (29)
Not reported	3 (5)
Possible or probable	15 (19)
Female	10 (67)
Male	5 (33)
Not reported	0 (0)

Abbreviation: IQR, interquartile range.

**TABLE 3:** Conditions associated with mixed AIHA in the 81 included patients.

Associated conditions	No. patients (% of total)^a^
Autoimmune disease	29 (36)
SLE	14 (48)
Evan's syndrome	5 (17)
Chronic AIHA (distinct from mixed AIHA)	2 (7)
Autoimmune vasculitis	1 (3)
Hypothyroidism	1 (3)
ITP	1 (3)
Kawasaki disease	1 (3)
MCTD	1 (3)
Primary biliary cholangitis	1 (3)
Sjogren's syndrome	1 (3)
Thyrotoxicosis	1 (3)
Infections	10 (12)
COVID‐19	3 (30)
EBV or CMV infection	2 (20)
HIV	2 (20)
Infectious mononucleosis (possible)	1 (10)
Chicken pox	1 (10)
Viral infection	1 (10)
Malignancy	17 (21)
Hematologic	14 (82)
Angioimmunoblastic T‐cell lymphoma	3 (21)
Non‐Hodgkin lymphoma (type not specified)	3 (21)
Waldenstrom macroglobulinemia	3 (21)
Chronic myeloid leukemia	2 (14)
Monoclonal B‐cell lymphocytosis	1 (7)
Hodgkin disease	1 (7)
T‐cell lymphoma (not further specified)	1 (7)
Solid tumor	3 (18)
Post‐transplantation	6 (7)
Hematopoietic progenitor cell	5 (83)
Solid organ	1 (17)
Other	4 (5)
α‐methyldopa therapy	1 (25)
CKD	1 (25)
Fanconi anemia	1 (25)
Pregnancy	1 (25)
None	18 (22)
Not reported	5 (6)

Abbreviations: AIHA, autoimmune hemolytic anemia; CKD, chronic kidney disease; CMV, cytomegalovirus; EBV, Epstein–Barr virus; HIV, human immunodeficiency virus; ITP, immune thrombocytopenia; MCTD, mixed connective tissue disease; SLE, systemic lupus erythematosus.

^a^ A single patient may have had more than one condition (e.g., hematologic malignancy and hematopoietic progenitor cell transplantation).

### 3.3 Laboratory and Immunohematology Findings

Initial hemoglobin values were reported for 47 patients, with a median of 5.7 g/dL (IQR: 4.7–6.9 g/dL) and nadir hemoglobin was reported for 41 patients (median 5.6 g/dL, IQR: 4.3–6.7 g/dL) (Table 4). Median initial and nadir hemoglobin values were similar for patients irrespective of outcome. There was no difference in the median initial hemoglobin (5.7 g/dL [IQR: 4.7–7.0 g/dL], 95% CI: 5.1–6.4 g/dL vs. 5.8 g/dL [IQR: 3.9–7.0 g/dL], 95% CI: 2.8–7.2 g/dL; *p* = 0.96) or the median hemoglobin nadir (5.6 g/dL [IQR: 4.4–6.8 g/dL], 95% CI: 4.8–6.6 g/dL vs. 5.1 g/dL [IQR: 3.7–6.7 g/dL], 95% CI: 3.2–7.0 g/dL; *p* = 0.44) between patients with “definite” and “possible or probable” mixed AIHA.

**TABLE 4:** Laboratory values for the 81 included patients.

Laboratory value (*n* = number of patients with that outcome and laboratory value reported)	Median (IQR), g/dL
Definite	
Initial hemoglobin (*n* = 34)	5.7 (4.7–7.0)
Hemoglobin nadir (*n* = 31)	5.6 (4.4–6.8)
Possible or probable	
Initial hemoglobin (*n* = 13)	5.8 (3.9–7.0)
Hemoglobin nadir (*n* = 10)	5.1 (3.7–6.7)
Initial hemoglobin, all patients (*n* = 47)	5.7 (4.7–6.9)
Remission (*n* = 24)	5.7 (5.0–7.0)
Chronic hemolysis (*n* = 16)	6.0 (4.7–7.4)
Deceased (*n* = 3)	5.9 (5.0–6.4)
Hemoglobin nadir, all patients (*n* = 41)	5.6 (4.3–6.7)
Remission (*n* = 19)	5.6 (4.1–6.8)
Chronic hemolysis (*n* = 18)	5.6 (4.5–6.5)
Deceased (*n* = 2)	5.0 (2.5–7.4)

Abbreviation: IQR, interquartile range.

All patients had a positive DAT for IgG and C3 per our definition of mixed AIHA. Cold agglutinin titers at 4°C were reported for 46 patients with a median of 64 (IQR: 8–256). Titers ranged from < 1 (i.e., neat or straight patient plasma without being diluted) to 8192, but 41.3% (19/46) were < 64. Among the subset with “definite” mixed AIHA, the titer was reported for 33 patients with a median of 32 (IQR: 8–128). Among the 73 patients with available thermal amplitude results, the cold agglutinin was reactive at ≥ 30°C in all patients, while it was explicitly stated that it reacted at 37°C in 31 patients.

### 3.4 Therapies and Outcomes

A total of 51 patients received at least one RBC transfusion; however, it was not specified whether most of the remaining patients required transfusion (i.e., it is not known whether they received RBC transfusion). Among the patients who had reported transfusions, 47.1% (24/51) went into remission, 33.3% (17/51) experienced chronic hemolysis, 11.8% (6/51) died, 5.9% (3/51) did not have outcomes reported, and 2.0% (1/51) were lost to follow‐up.

The number of RBC units was reported for 14 of the 51 patients in which it was explicitly stated that they received a transfusion, with these patients requiring a median of 7 units (IQR 5–10 units; range 2–33 units). Among these, 64.3% (9/14) went into remission, 21.4% (3/14) experienced chronic hemolysis, 7.1% (1/14) were lost to follow up, and 7.1% (1/14) did not have outcomes reported. When comparing those who went into remission and those who did not, there was no significant difference in the median number of RBC units required (8 units [IQR: 6–20 units], 95% CI: 5–29 units vs. 8 units [IQR: 4–8 units], 95% CI: 4–8 units; *p* = 0.58). However, a greater proportion of patients who went into remission received multiple therapies compared to those who did not go into remission (88.9%, 8/9 vs. 33.3%, 1/3).

Specific therapies were reported for 93.8% (76/81) of patients (Table 5); among these, corticosteroids (98.7%, 75/76), cyclophosphamide (17.1%, 13/76), rituximab (13.2%, 10/76), and intravenous immune globulin (IVIG) (11.8%, 9/76) were the most commonly employed therapeutic agents, though a wide range of treatments and treatment combinations were utilized. Corticosteroids were used alone in 44.7% (34/76) of patients. In this group, of the 30 patients with reported outcomes, 40.0% (12/30) went into remission from their mixed AIHA. Conversely, 53.3% (16/30) suffered from chronic hemolysis, 3.3% (1/30) died from severe hemolysis, and 3.3% (1/30) died from sepsis during treatment for underlying lymphoma, which was believed to be a contributing factor to the mixed AIHA.

**TABLE 5:** Therapies utilized for the 81 patients with mixed AIHA.

Therapy	No. patients (% of total)
Corticosteroids alone	34 (42)
Corticosteroids, rituximab	5 (6)
Corticosteroids, splenectomy	4 (5)
Corticosteroids, cyclophosphamide	3 (4)
Corticosteroids, IVIG	3 (4)
Corticosteroids, azathioprine, splenectomy	2 (2)
Corticosteroids, azathioprine	2 (2)
Corticosteroids and IVIG + lymphoma treatment (cyclophosphamide, doxorubicin, vincristine, prednisone)	2 (2)
Corticosteroids and cyclophosphamide + lymphoma treatment (cyclophosphamide, doxorubicin, vincristine, prednisone)	1 (1)
Corticosteroids, cyclophosphamide, and TPE + lymphoma treatment (cyclophosphamide, vincristine, prednisolone, and doxorubicin)	1 (1)
Etoposide, ifosfamide, and prednisolone	1 (1)
Corticosteroids, azathioprine, cyclophosphamide, splenectomy	1 (1)
Corticosteroids, TPE	1 (1)
Corticosteroids, mizoribine	1 (1)
Corticosteroids, chlorambucil, TPE	1 (1)
Corticosteroids, hydroxychloroquine, cyclophosphamide	1 (1)
Corticosteroids, azathioprine, chlorambucil	1 (1)
Corticosteroids, azathioprine, danazol	1 (1)
Corticosteroids, cyclophosphamide, vincristine	1 (1)
Corticosteroids + lymphoma treatment (cyclophosphamide, vincristine, prednisone)	1 (1)
Corticosteroids, cyclosporine, mycophenolate, IVIG	1 (1)
Corticosteroids, cyclophosphamide, vincristine, IVIG, rituximab, splenectomy	1 (1)
Corticosteroids, IVIG, cyclosporine, cyclophosphamide, splenectomy	1 (1)
Corticosteroids, IVIG, rituximab	1 (1)
Corticosteroids, IVIG, rituximab, mycophenolate mofetil, cyclophosphamide, TPE (×7), splenectomy	1 (1)
Corticosteroids, rituximab, tocilizumab	1 (1)
Corticosteroids, rituximab, TPE (×5), IVIG	1 (1)
Corticosteroids, vincristine, cyclophosphamide	1 (1)
Tocilizumab	1 (1)
Not reported	5 (6)

Abbreviations: AIHA, autoimmune hemolytic anemia; IVIG, intravenous immune globulin; TPE, therapeutic plasma exchange.

A minimum of two therapeutic interventions was used in 53.9% (41/76) of patients; the specific therapies and combinations varied based on factors including disease status, underlying comorbidities, and the timeframe over which the cases occurred. Overall, 51.2% (21/41) of patients who received ≥ 2 therapeutic interventions went into remission, while 34.1% (14/41) experienced chronic hemolysis and 14.6% (6/41) died. All five of the patients that received a regimen of corticosteroids and rituximab achieved remission. Ten patients underwent splenectomy, six of whom continued to experience chronic hemolysis, two went into remission, and two died.

Overall, outcomes were reported for 91.4% (74/81) patients (Table 6); remission occurred in 43.2% (35/81) of patients, while 37.0% (30/81) experienced chronic hemolysis and 11.1% (9/81) died. Of the 35 patients who went into remission, 60.0% (21/35) required more than one therapeutic intervention. Among patients with “definite” mixed AIHA, 40.9% (27/66) had chronic hemolysis, 39.4% (26/66) achieved remission, and 13.6% (9/66) died.

**TABLE 6:** Outcomes of 81 patients with mixed AIHA.

Outcome	No. patients (% of total)
Remission	35 (43.2)
Definite	26 (74.3)
Possible or probable	9 (25.7)
Chronic hemolysis	30 (37.0)
Definite	27 (90.0)
Possible or probable	3 (10.0)
Deceased, all	9 (11.1)
Sepsis	3 (33.3)
Hemolysis	2 (22.2)
Cause not reported	1 (11.1)
Malignancy	1 (11.1)
Cardiopulmonary arrest	1 (11.1)
Pneumonia	1 (11.1)
Deceased, definite	9 (11.1)
Deceased, possible or probable	0 (0.0)
Lost to follow up	1 (1.2)
Not reported	6 (7.4)
Definite	4 (66.7)
Possible or probable	2 (33.3)

Abbreviation: AIHA, autoimmune hemolytic anemia.

Among those with cold agglutinin titer data and clinical outcomes reported, there was no difference in the proportion of patients who went into remission with titers < 64 compared to those with titers ≥ 64 (62.5%, 10/16 vs. 58.3%, 14/24; *p* > 0.99).

## 4 Discussion

We identified 35 reports encompassing 81 patients who met strict criteria for mixed AIHA, with a significant overrepresentation of females. The severity of anemia was notable, with a median hemoglobin level below 6.0 g/dL, aligning with previous findings [1]. Treatment approaches were heterogeneous, and more than half of patients received at least two therapeutic interventions, with corticosteroids, rituximab, and cyclophosphamide among the most frequently used therapies. Fewer than half of the patients achieved remission, underscoring the complex nature and treatment challenges of this rare condition and emphasizing the need for standardized diagnostic and management strategies.

One of our key findings was the lack of a significant difference in remission rates independent of cold antibody titer, suggesting that cold agglutinin titer alone may not reliably predict disease severity or treatment response. Previous studies have emphasized the importance of thermal amplitude rather than titer levels alone in determining the pathogenicity of cold agglutinins [27, 28, 29]. However, many studies identified in the literature diagnosed mixed AIHA based solely on a positive DAT and red cell agglutination (e.g., such as at immediate spin or as observed on peripheral blood smear examination), precluding their inclusion in our analysis. The mere presence of a cold agglutinin does not establish pathogenicity [30]. Indeed, Petz and Garratty reported that 35% of patients with WAIHA have strong cold agglutinins that, when tested appropriately, display low titers at 4°C and are non‐reactive at physiologic body temperature (i.e., narrow thermal amplitude) [15], indicating a lack of clinical significance. Therefore, identifying a pathologic cold autoantibody that reacts at body temperature (≥ 30°C) appears to be a more relevant marker when clinical significance is in question.

The heterogeneity in therapeutic regimens across studies complicates efforts to develop an optimal treatment algorithm for mixed AIHA. However, corticosteroids were the most frequently used therapy, employed as monotherapy in 42% of cases—many of which were reported earlier in the study period. However, outcomes with corticosteroid monotherapy were suboptimal: only 40% of patients achieved remission, while 53% experienced chronic hemolysis, and 7% died due to hemolysis, treatment‐related complications, or their underlying disease. In contrast, approximately 51% of patients who received combination therapy achieved remission, including all five patients who received a regimen of corticosteroids plus rituximab. These findings align with prior studies in cold AIHA, which have shown limited efficacy of corticosteroids when used alone [31, 32]. This finding raises critical questions as to whether early combination therapy (and which particular agents) should be the first‐line treatment for mixed AIHA. Given the demonstrated efficacy of rituximab and other immunosuppressive agents in refractory AIHA cases [32, 33, 34, 35], their earlier integration into treatment regimens for patients with mixed AIHA may improve outcomes.

Additionally, almost two‐thirds (62%) of patients required RBC transfusion, though transfusion status and efficacy (i.e., how the patient responded to transfusion) were inconsistently reported. The median number of transfused RBC units was high (7 units, range 2–33), yet the specific number of units required was documented in only 14 reports. These findings suggest that a subset of patients with mixed AIHA experiences profound and persistent anemia. The significant transfusion burden raises concerns about alloimmunization, iron overload, and the potential for transfusion‐related complications, which should be carefully considered in clinical management. Interestingly, the need for RBC transfusion was not clearly associated with treatment outcomes, suggesting that transfusion burden alone may not reliably predict disease severity or ultimate remission, although it is important to note that many studies did not include details regarding transfusion threshold or other factors that may have influenced decisions related to transfusion.

## 5 Limitations

Our study has limitations, primarily related to the reliance on published case reports and small retrospective studies, which may be subject to publication bias and incomplete reporting. Further, the diagnostic heterogeneity (e.g., different testing platforms, methodologies, and reagents; various immunohematologic criteria for diagnosing mixed AIHA) across studies complicates direct comparisons. Many reports defined mixed AIHA based on a positive DAT for both IgG and complement, without stringent thermal amplitude testing or additional immunohematologic assessments, such as serum and elution studies, precluding further analysis. Acutely ill patients may exhibit a positive DAT that is not necessarily due to an autoantibody [36], making it essential to differentiate true mixed AIHA from incidental serologic findings. Given the variability in diagnostic approaches, the true prevalence and clinical impact of mixed AIHA remain uncertain. Although a strict framework was used to identify reviewed cases, the diagnosis of mixed AIHA remains controversial given the range of immunohematology and clinical findings described with the condition. Furthermore, the broad timeframe over which the included studies were published (1975–2024) poses challenges in comparing treatment strategies, as advancements in medical management have evolved significantly over time. Finally, while some patients with acute, severe disease may be treated with faster‐acting therapies including bendamustine, eculizumab, and therapeutic plasma exchange, our review did not separately analyze these patients due to small sample size and inconsistent reporting.

## 6 Future Research Directions

Future research efforts should focus on the development and implementation of standardized diagnostic criteria for mixed AIHA and the establishment of prospective, multicenter registries to better characterize this rare condition. Immunohematology evaluation should include characterization of both the warm and cold components, including serum and elution studies, cold agglutinin titers, and thermal amplitude testing [37, 38, 39, 40]. While antibody titer testing may be more widely available, titers are subject to wide inter‐ and intra‐laboratory variation [41], and future research should investigate the effect of changes in thermal amplitude over the course of therapy on clinical outcomes [27, 42].

Importantly, there is a pressing need for clinical recommendations that guide a *standardized diagnostic approach*, not only to improve case definitions for research but also to inform treatment selection and monitor therapeutic response. Diagnostic inconsistencies across studies have contributed to challenges in interpreting treatment efficacy, as the lack of detailed immunohematologic characterization obscures whether observed outcomes reflect true therapeutic effects or underlying diagnostic heterogeneity. Future recommendations should emphasize incorporating comprehensive immunohematology testing into both initial and longitudinal patient evaluations, ideally with defined testing protocols that can be adopted across institutions. Without such diagnostic rigor, clinicians and researchers cannot reliably determine whether treatments are effective or ineffective, hindering both evidence synthesis and patient care. Establishing clear diagnostic benchmarks will enhance the interpretability of future studies and may facilitate biomarker development to predict treatment response.

Another notable consideration is the underlying immunopathologic mechanisms that contribute to mixed AIHA. The concurrent presence of warm‐reactive IgG and cold‐reactive IgM autoantibodies suggests complex immune dysregulation involving both T‐cell‐dependent and independent mechanisms. Warm‐reactive IgG antibodies are typically associated with T‐cell‐mediated loss of self‐tolerance [43], whereas cold‐reactive IgM antibodies are often associated with monoclonal or oligoclonal B‐cell processes, in conjunction with innate immunity and complement activation [44]. Improved understanding of these immunologic pathways may guide the development of targeted therapeutic interventions, such as therapies that modulate T‐cell pathways.

Finally, since patients with mixed AIHA in this study frequently required multiple lines of therapy, future prospective studies should evaluate whether initial combination therapy (e.g., corticosteroids plus rituximab) [17, 45] offers superior long‐term disease control compared to a sequential escalation approach. Similarly, novel therapeutic agents, including complement inhibitors (e.g., sutimlimab, pegcetacoplan) and other targeted therapies (e.g., splenic tyrosine kinase [syk] inhibitors, Bruton tyrosine kinase [BTK] inhibitors, neonatal Fc receptor inhibitors, etc.) [46, 47, 48, 49, 50, 51, 52, 53, 54], warrant investigation for their potential role in treating mixed AIHA. Given the high rate of disease morbidity and mortality (11%) observed in our study, exploring treatment strategies beyond conventional immunosuppression is essential for improving patient outcomes.

## 7 Conclusion

Mixed AIHA is a severe and often refractory subtype of AIHA, characterized by high rates of chronic hemolysis, substantial transfusion burden, and mortality. Our findings emphasize the urgent need for refined diagnostic criteria, early incorporation of combination therapy, and focused research into innovative treatment strategies. Prospective clinical studies designed to identify optimal management approaches are essential to enhancing clinical outcomes and improving quality of life for patients affected by this challenging hematologic condition.

## Ethics Statement

The authors have nothing to report.

## Consent

The authors have nothing to report.

## Conflicts of Interest

E.M.B. reports personal fees and non‐financial support from Grifols, Abbott, UptoDate, Tegus, and Health Advances outside of the submitted work. E.M.B. is a co‐investigator on a US government funded clinical trial evaluating Mirasol Pathogen Reduction Technology. E.M.B. is a member of the U.S. Food and Drug Administration (FDA) Blood Products Advisory Committee. Any views or opinions expressed in this manuscript are his and are based on his own scientific expertise and professional judgment; they do not necessarily represent the views of the Blood Products Advisory Committee or the formal position of the FDA and also do not bind or otherwise obligate or commit either the Advisory Committee or the FDA to the views expressed. The other authors declare no conflicts of interest.

## Supporting information


**Data S1.** Supporting Information.

## Data Availability

For data requests, please contact Dr. Jeremy W. Jacobs at jeremy.w.jacobs@vumc.org.
