# Supplementary material for: Mixed Autoimmune Hemolytic Anemia: A Systematic Review of Epidemiology, Clinical Characteristics, Therapies, and Outcomes
Source: Am J Hematol. 2025 May 20;100(8):1397–407. doi: 10.1002/ajh.27721 (PMC12232602; doi:10.1002/ajh.27721)
Supplement: Supplementary file 1 — Data S1. Supporting Information. [file AJH-100-1397-s001.docx]

**Mixed Autoimmune Hemolytic Anemia: A Systematic Review of Epidemiology, Clinical Characteristics, Therapies, and Outcomes**

**Page 2:** Supplemental Figure 1. Number of patients reported by article publication year

**Pages 3-5:** Supplemental Table 1. All studies included in the analysis

**Pages 6-8:** Literature search strategies

**Supplemental Figure 1.** Number of patients reported by article publication year

**Supplemental Table 1.** All studies included in the analysis

| **Reference** | **Patients included in our analysis (no.)** |
| --- | --- |
| Ahmad I, Majeed Memon A, Moiz B. Mixed IgM cold and IgG warm autoimmune hemolytic anemia complicated by acral gangrene. *Transfusion*. 2018;58(5):1093-1094. doi:10.1111/trf.14572 | 1 |
| Chinnadurai A, Strum S, Ghassemian A, Fortin D, Foster C, Breadner D. A Rare Association of Mixed Autoimmune Hemolytic Anemia with Gastric Carcinoma. *Case Rep Oncol*. 2023;16(1):1209-1216. doi:10.1159/000534278 | 1 |
| Crookston JH. Hemolytic anemia with IgG and IgM autoantibodies and allontibodies. *Arch Intern Med*. 1975;135(10):1314-1315. | 1 |
| Datta SS, Reddy M, Basu S, Krishnan S. Blood Group Discrepancy-First Sign of Autoimmune Hemolytic Anemia after Hematopoietic Stem Cell Transplantation in a Child. *Indian J Hematol Blood Transfus*. 2016;32(Suppl 1):211-213. doi:10.1007/s12288-015-0630-2 | 1 |
| De Angelis V, Biasinutto C, Pradella P, Errante D. Mixed-type auto-immune haemolytic anaemia in a patient with HIV infection. *Vox Sang*. 1995;68(3):191-194. doi:10.1111/j.1423-0410.1995.tb03926.x | 1 |
| Edwards BP, Senapati SG, Kasianchyk M, Shah J, Ayvali F, Maharaj S. Mixed autoimmune hemolytic anemia as the initial presentation of systemic lupus erythematosus: A case report and review. *EJHaem*. 2024;5(5):1053-1056. doi:10.1002/jha2.1008 | 1 |
| Freedman J, Lim FC, Musclow E, Fernandes B, Rother I. Autoimmune hemolytic anemia with concurrence of warm and cold red cell autoantibodies and a warm hemolysin. *Transfusion*. 1985;25(4):368-372. doi:10.1046/j.1537-2995.1985.25485273819.x | 1 |
| Freedman J, Newlands M. Autoimmune haemolytic anaemia with the unusual combination of both IgM and IgG autoantibodies. *Vox Sang*. 1977;32(2):61-68. doi:10.1111/j.1423-0410.1977.tb00607.x | 2 |
| Fuja C, Kothary V, Carll TC, Singh S, Mansfield P, Wool GD. Hyperhemolysis in a patient with sickle cell disease and recent SARS-CoV-2 infection, with complex auto- and alloantibody work-up, successfully treated with tocilizumab. *Transfusion*. 2022;62(7):1446-1451. doi:10.1111/trf.16932 | 1 |
| Granel B, Rossi P, Bernard F, et al. Good outcome after rituximab treatment for a mixed warm and cold autoimmune haemolytic anaemia. *BMJ Case Rep*. 2009;2009:bcr09.2008.0857. doi:10.1136/bcr.09.2008.0857 | 1 |
| Horn B, Viele M, Mentzer W, Mogck N, DeSantes K, Cowan M. Autoimmune hemolytic anemia in patients with SCID after T cell-depleted BM and PBSC transplantation. *Bone Marrow Transplant*. 1999;24(9):1009-1013. doi:10.1038/sj.bmt.1702011 | 1 |
| Hsu HC, Lin CK, Chau WK, Hu HY, Liu SM. Combined cold- and warm-antibody autoimmune hemolytic anemia--review of the literature and a case report. *Gaoxiong Yi Xue Ke Xue Za Zhi*. 1989;5(6):350-356. | 1 |
| Jacobs J, Eichbaum Q. COVID-19 associated with severe autoimmune hemolytic anemia. *Transfusion*. 2021;61(2):635-640. doi:10.1111/trf.16226 | 1 |
| Kajii E, Miura Y, Ikemoto S. Characterization of autoantibodies in mixed-type autoimmune hemolytic anemia. *Vox Sang*. 1991;60(1):45-52. doi:10.1111/j.1423-0410.1991.tb00870.x | 3 |
| Kanie T, Higuchi T, Koyamada R, Suda M, Okada S. Very elderly-onset systemic lupus erythematosus presented with mixed-type autoimmune hemolytic anemia. *Aging Med Healthc*. 2017;8(1):40-43. doi:10.33879/AMH.2017.1711. | 1 |
| Kimura S, Osaki M, Sakai S, et al. *Nihon Ronen Igakkai Zasshi*. 2019;56(3):331-335. doi:10.3143/geriatrics.56.331 | 1 |
| Klassen S, Wang M, Ross C, Verhovsek M. Refractory mixed autoimmune hemolysis, thrombocytopenia, and thrombosis: a diagnostic puzzle. *Ann Hematol*. 2015;94(6):1055-1056. doi:10.1007/s00277-014-2292-9 | 1 |
| Koduri PR, Singa P, Nikolinakos P. Autoimmune hemolytic anemia in patients infected with human immunodeficiency virus-1. *Am J Hematol*. 2002;70(2):174-176. doi:10.1002/ajh.10096 | 1 |
| Li M, Goldfinger D, Yuan S. Autoimmune hemolytic anemia in pediatric liver or combined liver and small bowel transplant patients: a case series and review of the literature. *Transfusion*. 2012;52(1):48-54. doi:10.1111/j.1537-2995.2011.03254.x | 2 |
| Mayer B, Yürek S, Kiesewetter H, Salama A. Mixed-type autoimmune hemolytic anemia: differential diagnosis and a critical review of reported cases. *Transfusion*. 2008;48(10):2229-2234. doi:10.1111/j.1537-2995.2008.01805.x | 2 |
| McCann EL, Shirey RS, Kickler TS, Ness PM. IgM autoagglutinins in warm autoimmune hemolytic anemia: a poor prognostic feature. *Acta Haematol*. 1992;88(2-3):120-125. doi:10.1159/000204666 | 5 |
| Moake JL, Schultz DR. Hemolytic anemia associated with multiple autoantibodies and low serum complement. *Am J Med*. 1975;58(3):431-437. doi:10.1016/0002-9343(75)90610-5 | 1 |
| Mohd Shukri N, Ab Aziz NAFAA. Mixed autoimmune haemolytic anaemia in a COVID-19 patient. *Malays J Pathol*. 2023;45(1):135-138. | 1 |
| Morselli M, Luppi M, Potenza L, et al. Mixed warm and cold autoimmune hemolytic anemia: complete recovery after 2 courses of rituximab treatment. *Blood*. 2002;99(9):3478-3479. doi:10.1182/blood-2002-01-0018 | 1 |
| Panzarino V, Estrada J, Benson K, Postoway N, Garratty G. Autoimmune hemolytic anemia after Kawasaki disease in a child. *Int J Hematol*. 1993;57(3):259-263. | 1 |
| Qiao L, Chen J, Leng XM, et al. Agranulocytosis and mixed-type autoimmune hemolytic anemia in primary sjögren's syndrome: a case report and review of the literature. *Int J Rheum Dis*. 2016;19(12):1351-1353. doi:10.1111/1756-185X.12803 | 1 |
| Rai P, Sharma G, Singh D, Garg J. Rare presentation of mixed autoimmune hemolytic anemia in children: Report of 2 cases. *J Lab Physicians*. 2017;9(4):332-336. doi:10.4103/JLP.JLP_95_17 | 2 |
| Rokicka M, Styczynski J, Michalewska B, et al. Fatal combined immune hemolytic anemia after double cord blood transplantation in imatinib-resistant CML. *Bone Marrow Transplant*. 2009;44(6):383-385. doi:10.1038/bmt.2009.25 | 1 |
| Sharma G, More S, Rajput S, Sharma N, Choudhary S, Chatterjee T. Challenges in the diagnosis and management of autoimmune hemolytic anemia: A case-based approach. Experience from a tertiary care hospital in the Haryana region. *Indian J Pathol Microbiol*. 2024;67(4):801-806. doi:10.4103/ijpm.ijpm_896_23 | 2 |
| Shulman IA, Branch DR, Nelson JM, Thompson JC, Saxena S, Petz LD. Autoimmune hemolytic anemia with both cold and warm autoantibodies. *JAMA*. 1985;253(12):1746-1748. | 12 |
| Sokol RJ, Hewitt S, Stamps BK. Autoimmune hemolysis: mixed warm and cold antibody type. *Acta Haematol*. 1983;69(4):266-274. doi:10.1159/000206903 | 24 |
| Suzuki E, Kanno T, Saito Y, Shimbo T. Systemic Lupus Erythematosus and Antiphospholipid Syndrome Accompanied by Mixed-Type Autoimmune Hemolytic Anemia. *Case Rep Rheumatol*. 2023;2023:4963196. doi:10.1155/2023/4963196 | 1 |
| Tanaka Y, Masuya M, Katayama N, et al. Development of mixed-type autoimmune hemolytic anemia and Evans' syndrome following chicken pox infection in a case of low-titer cold agglutinin disease. *Int J Hematol*. 2006;84(3):220-223. doi:10.1532/IJH97.06046 | 1 |
| Turudic D, Dejanovic Bekic S, Mucavac L, Pavlovic M, Milosevic D, Bilic E. Case report: Autoimmune hemolytic anemia caused by warm and cold autoantibodies with complement activation-etiological and therapeutic issues. *Front Pediatr*. 2023;11:1217536. doi:10.3389/fped.2023.1217536 | 1 |
| Win N, Tiwari D, Keevil VL, Needs M, Lakhani A. Mixed-type autoimmune haemolytic anaemia: unusual cases and a case associated with splenic T-cell angioimmunoblastic non-Hodgkin's lymphoma. *Hematology*. 2007;12(2):159-162. doi:10.1080/110245330601111466 | 2 |

**Literature search strategies**

**PubMed (NCBI)**

Date of Search: September 30, 2024

Number of results: 847

| **Search** | **Query** |
| --- | --- |
| 1 | "Anemia, Hemolytic, Autoimmune"[Mesh] |
| 2 | AIHA[tiab] |
| 3 | autoimmune haemolysis[tiab] OR autoimmune hemolysis[tiab] |
| 4 | autoimmune haemolytic anaemia*[tiab] OR autoimmune haemolytic anemia*[tiab] |
| 5 | autoimmune hemolytic anaemia[tiab] OR autoimmune hemolytic anemia*[tiab] |
| 6 | cold agglutinin disease*[tiab] OR cold antibody disease*[tiab] |
| 7 | cold antibody haemolytic anaemia*[tiab] OR cold antibody haemolytic anemia*[tiab] |
| 8 | cold antibody hemolytic anaemia*[tiab] OR cold antibody hemolytic anemia*[tiab] |
| 9 | immune mediated anaemia*[tiab] OR immune mediated anemia*[tiab] |
| 10 | OR/ 1-9 |
| 11 | Mixed[tiab] OR combined[tiab] |
| 12 | warm[tiab] AND cold[tiab] |
| 13 | IgM[tiab] AND IgG[tiab] |
| 14 | immunoglobulin M[tiab] AND immunoglobulin G[tiab] |
| 15 | OR/ 11-14 |
| 16 | 10 AND 15 |

**Cochrane Central Register of Controlled Trials - CENTRAL (Wiley)**

Date of Search: September 30, 2024

Number of results: 105

| **Search** | **Query** |
| --- | --- |
| 1 | (aiha OR autoimmune haemolysis OR autoimmune haemolytic anaemia* OR autoimmune haemolytic anemia* OR autoimmune hemolysis OR autoimmune hemolytic anaemia OR autoimmune hemolytic anemia* OR cold agglutinin disease* OR cold antibody disease* OR cold antibody haemolytic anaemia* OR cold antibody haemolytic anemia* OR cold antibody hemolytic anaemia* OR cold antibody hemolytic anemia* OR immune mediated anaemia* OR immune mediated anemia*):ti,ab,kw |
| 2 | (mixed OR combined):ti,ab,kw |
| 3 | (warm AND cold):ti,ab,kw |
| 4 | (igm AND igg):ti,ab,kw |
| 5 | (“immunoglobulin m” AND “immunoglobulin g”):ti,ab,kw |
| 6 | #2 OR #3 OR #4 OR #5 |
| 7 | #1 AND #6 |

**Cochrane Database of Systematic Reviews - CDSR (Wiley)**

Date of Search: September 30, 2024

Number of results: 2

| **Search** | **Query** |
| --- | --- |
| 1 | (aiha OR autoimmune haemolysis OR autoimmune haemolytic anaemia* OR autoimmune haemolytic anemia* OR autoimmune hemolysis OR autoimmune hemolytic anaemia OR autoimmune hemolytic anemia* OR cold agglutinin disease* OR cold antibody disease* OR cold antibody haemolytic anaemia* OR cold antibody haemolytic anemia* OR cold antibody hemolytic anaemia* OR cold antibody hemolytic anemia* OR immune mediated anaemia* OR immune mediated anemia*):ti,ab,kw |
| 2 | (mixed OR combined):ti,ab,kw |
| 3 | (warm AND cold):ti,ab,kw |
| 4 | (igm AND igg):ti,ab,kw |
| 5 | (“immunoglobulin m” AND “immunoglobulin g”):ti,ab,kw |
| 6 | #2 OR #3 OR #4 OR #5 |
| 7 | #1 AND #6 |

**Embase (Elsevier)**

Date of Search: September 30, 2024

Number of results: 884

| **Search** | **Query** |
| --- | --- |
| 1 | 'autoimmune hemolytic anemia'/exp |
| 2 | 'aiha':ti,ab |
| 3 | 'autoimmune haemolysis':ti,ab OR 'autoimmune hemolysis':ti,ab |
| 4 | 'autoimmune haemolytic anaemia*':ti,ab OR 'autoimmune haemolytic anemia*':ti,ab |
| 5 | 'autoimmune hemolytic anaemia':ti,ab OR 'autoimmune hemolytic anemia*':ti,ab |
| 6 | 'cold agglutinin disease*':ti,ab OR 'cold antibody disease*':ti,ab |
| 7 | 'cold antibody haemolytic anaemia*':ti,ab OR 'cold antibody haemolytic anemia*':ti,ab |
| 8 | 'cold antibody hemolytic anaemia*':ti,ab OR 'cold antibody hemolytic anemia*':ti,ab |
| 9 | 'immune mediated anaemia*':ti,ab OR 'immune mediated anemia*':ti,ab |
| 10 | OR/ #1-9 |
| 11 | 'mixed':ti,ab OR 'combined':ti,ab |
| 12 | 'warm':ti,ab AND 'cold':ti,ab |
| 13 | 'igm':ti,ab AND 'igg':ti,ab |
| 14 | 'immunoglobulin m':ti,ab AND 'immunoglobulin g':ti,ab |
| 15 | OR/ #11-14 |
| 16 | #10 AND #15 |
| 17 | #16 AND ([article]/lim OR [article in press]/lim OR [preprint]/lim) |

**Scopus (Elsevier)**

Date of Search: September 30, 2024

Number of results: 772

| **Search** | **Query** |
| --- | --- |
| 1 | TITLE-ABS(“aiha” ) |
| 2 | TITLE-ABS(“autoimmune haemolysis” OR “autoimmune hemolysis”) |
| 3 | TITLE-ABS(“autoimmune haemolytic anaemia*” OR “autoimmune haemolytic anemia*”) |
| 4 | TITLE-ABS(“autoimmune hemolytic anaemia” OR “autoimmune hemolytic anemia*”) |
| 5 | TITLE-ABS(“cold agglutinin disease*” OR “cold antibody disease*”) |
| 6 | TITLE-ABS(“cold antibody haemolytic anaemia*” OR “cold antibody haemolytic anemia*”) |
| 7 | TITLE-ABS(“cold antibody hemolytic anaemia*” OR “cold antibody hemolytic anemia*”) |
| 8 | TITLE-ABS(“immune mediated anaemia*” OR “immune mediated anemia*”) |
| 9 | OR/ 1-8 |
| 10 | TITLE-ABS("mixed" OR "combined") |
| 11 | TITLE-ABS("warm" AND "cold") |
| 12 | TITLE-ABS("igm" AND "igg") |
| 13 | TITLE-ABS("immunoglobulin m" AND "immunoglobulin g") |
| 15 | OR/ 10-13 |
| 15 | 9 AND 14 |
| 16 | 15 LIMIT-TO ( DOCTYPE , "ar" ) OR LIMIT-TO ( DOCTYPE , "re" ) |

**Web of Science (Clarivate)**

Date of Search: September 30, 2024

Number of results: 1158

| **Search** | **Query** |
| --- | --- |
| 1 | TS=(aiha ) |
| 2 | TS=(autoimmune haemolysis OR autoimmune hemolysis) |
| 3 | TS=(autoimmune haemolytic anaemia* OR autoimmune haemolytic anemia*) |
| 4 | TS=(autoimmune hemolytic anaemia OR autoimmune hemolytic anemia*) |
| 5 | TS=(cold agglutinin disease* OR cold antibody disease*) |
| 6 | TS=(cold antibody haemolytic anaemia* OR cold antibody haemolytic anemia*) |
| 7 | TS=(cold antibody hemolytic anaemia* OR cold antibody hemolytic anemia*) |
| 8 | TS=(immune mediated anaemia* OR immune mediated anemia*) |
| 9 | OR/ 1-8 |
| 10 | TS=(mixed OR combined) |
| 11 | TS=(warm AND cold) |
| 12 | TS=(igm AND igg) |
| 13 | TS=(immunoglobulin m AND immunoglobulin g) |
| 14 | OR/ 10-13 |
| 15 | 9 AND 14 |
| 16 | 15 AND (Article or Review Article or Early Access (Document Types)) |
